# Metabolic regulation of immune responses to cancer

**DOI:** 10.20892/j.issn.2095-3941.2022.0381

**Published:** 2022-10-24

**Authors:** Jannis Wißfeld, Anke Werner, Xin Yan, Nora ten Bosch, Guoliang Cui

**Affiliations:** 1 Helmholtz Institute for Translational Oncology (HI-TRON), Mainz 55131, Germany; 2 T Cell Metabolism Group (D192), German Cancer Research Center (DKFZ), Heidelberg 69120, Germany; 3 Faculty of Biosciences, Heidelberg University, Heidelberg 69120, Germany

**Keywords:** Lipids, amino acids, cancer, anti-tumor immunity, T cells, NK cells, metabolism, immunometabolism

## Abstract

The tumor microenvironment is an ecosystem composed of multiple types of cells, such as tumor cells, immune cells, and cancer-associated fibroblasts. Cancer cells grow faster than non-cancerous cells and consume larger amounts of nutrients. The rapid growth characteristic of cancer cells fundamentally alters nutrient availability in the tumor microenvironment and results in reprogramming of immune cell metabolic pathways. Accumulating evidence suggests that cellular metabolism of nutrients, such as lipids and amino acids, beyond being essential to meet the bioenergetic and biosynthetic demands of immune cells, also regulates a broad spectrum of cellular signal transduction, and influences immune cell survival, differentiation, and anti-tumor effector function. The cancer immunometabolism research field is rapidly evolving, and exciting new discoveries are reported in high-profile journals nearly weekly. Therefore, all new findings in this field cannot be summarized within this short review. Instead, this review is intended to provide a brief introduction to this rapidly developing research field, with a focus on the metabolism of two classes of important nutrients—lipids and amino acids—in immune cells. We highlight recent research on the roles of lipids and amino acids in regulating the metabolic fitness and immunological functions of T cells, macrophages, and natural killer cells in the tumor microenvironment. Furthermore, we discuss the possibility of “editing” metabolic pathways in immune cells to act synergistically with currently available immunotherapies in enhancing anti-tumor immune responses.

## Introduction

In recent decades, cancer research has made substantial progress in the understanding of cancer biology and the development of therapy approaches against specific cancers. Scientists from the German Cancer Research Center (DKFZ) have been major drivers in this process. In 1989, a research group from DKFZ found that monoclonal antibodies targeting APO-1 (also known as CD95 or FasR) on human lymphoma cells trigger apoptosis of lymphoma cells *in vivo* and induce regression of lymphoma in a mouse model^1^. A broad spectrum of tumor cells express the ligand of APO-1, APO-1L (also known as CD95L, CD178, and FasL), which induces apoptosis of APO-1-expressing lymphocytes *in vitro*, in a process called tumor counterattack. *In vivo*, the interaction between APO-1 and APO-1L delays tumor growth in a neutrophil-independent manner^2^. More recent approaches target mechanisms of T cell exhaustion, a term used interchangeably with T cell dysfunction herein, and enhance anti-tumor immunity by using immune checkpoint blockade-based treatments. The most frequent targets are CTLA-4, PD-1, and its ligand PD-L1^3,4^. In addition, combinatory treatments such as anti-CD40 and an inhibitor of MAPK and ERK have been found to be promising candidates. This polytherapy synergistically suppresses Kras mutation-driven pancreatic ductal adenocarcinoma^5^. Moreover, inhibition of C-X-C motif chemokine 12 (CXCL12) has been found to promote T cell accumulation and to act synergistically with checkpoint inhibitors, thus providing clinical benefits to patients with advanced stage pretreated metastatic colorectal and pancreatic cancer in a phase I/II trial^6^. Furthermore, IL-10 prevents excessive activation-induced exhaustion of CD8^+^ T cells in a model of chronic lymphocytic leukemia and delays the development of leukemia^7^. Finally, our group has found that T cells express regulator of G-protein signaling (Rgs)-16 at high levels. *Rgs16* deficiency inhibits CD8^+^ T cell apoptosis and acts synergistically with PD-1 blockade in enhancing anti-tumor CD8^+^ T cell responses. Human *RGS16* mRNA expression levels in the CD8^+^ tumor-infiltrating T cells of patients with melanoma negatively correlate with the expression of genes associated with T cell stemness and are predictive of low responses to PD-1 blockade^8^.

However, checkpoint blockade therapy approaches have been only partially successful. Consequently, the influence of the tumor microenvironment (TME) on anti-tumor immunity and the field of immunometabolism have become a focus. Tumor-derived metabolic stimuli shape the TME into an immunosuppressive region, thereby hampering therapeutic approaches. In a mouse model of non-viral hepatocellular carcinoma (HCC), nonalcoholic steatohepatitis has been found to induce HCC. Liver-resident CD8^+^ T cells are sensitive to metabolic stimuli and are aberrantly activated by anti-PD-1 treatment. These auto-reactive CD8^+^PD-1^+^ T cells cause tissue damage instead of leading to HCC regression. Depleting CD8^+^ T cells or neutralizing TNFα has been found to ameliorate HCC progression in mice receiving anti-PD1, thus suggesting a rationale for stratifying patients with HCC according to etiology before immune checkpoint-based immunotherapy^9,10^. Kupffer cell-derived reactive oxygen species and TNFα promote cholangiocellular proliferation and oncogenic transformation. Depletion of Kupffer cells or blocking the TNFα signaling pathway decreases cholangiocellular oncogenic transformation^11^. Furthermore, macrophage-derived NO is required for the expression of vessel adhesion molecules, which are required for T cell extravasation and infiltration into tumors^12^. Interestingly, alterations of nutrient availability in the TME have also been shown to affect anti-tumor immunity. These alterations can be triggered by genetic mutations, which reprogram tumor cell metabolic pathways^13^. Our laboratory has found that CD8^+^ T cells in tumors increase the uptake of oxidized low-density lipoprotein (oxLDL) in a CD36-dependent manner. OxLDL promotes CD8^+^ T cell exhaustion through lipid peroxidation^14^. Moreover, the amino acid tryptophan is catabolized by tryptophan-2,3-dioxygenase and indoleamine 2,3 dioxygenase (IDO) in human tumor cells into kynurenine, an endogenous ligand of the transcription factor aryl hydrocarbon receptor (AHR). Kynurenine potently suppresses antitumor immune responses^15^, as extensively discussed in a later section herein. A recent study has revealed that the L-amino acid oxidase interleukin-4-induced-1 (IL4I1) generates indole metabolites and kynurenic acid, which are agonists of AHR, thereby promoting cancer cell motility and suppressing adaptive immunity. Because IDO inhibitors do not block IL4I1, these findings may explain the failure of a phase III clinical trial combining immune checkpoint blockade with IDO1 inhibition for cancer treatment^16^. An additional important finding of DKFZ scientists was that tumor cells with mutations in the isocitrate dehydrogenase gene produce the oncometabolite (R)-2-hydroxyglutarate, which inhibits immune responses and promotes tumor immunosuppression in the TME^17^.

These recent advances in immunometabolism research underscore the importance of understanding how the altered availability of nutrients, including glucose, lipids, and amino acids, in the TME changes anti-tumor immunity. In this review, we discuss the influence of changes in lipid and amino acid content within the TME on anti-tumor immunity. The role of glucose metabolism on immune responses to cancer has been extensively reviewed elsewhere and thus will not be discussed here^18–22^.

## Lipid metabolism regulates immune responses to tumors

**Figure 1:**
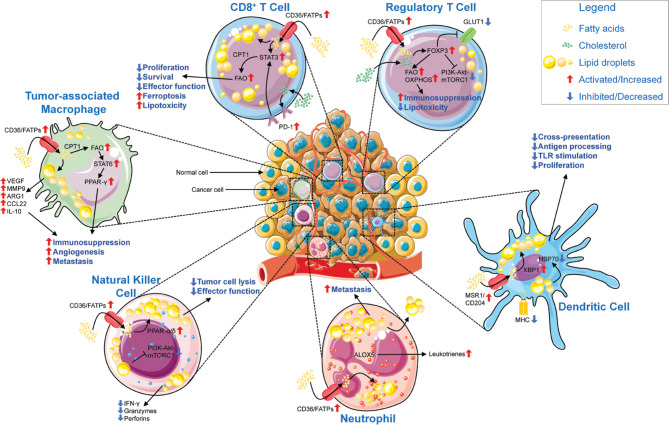
Lipid metabolism impairs anti-tumor immunity. In the lipid-rich TME, infiltrating CD8^+^ T cells upregulate CD36 or FATPs, thereby increasing lipid uptake. Lipid accumulation and subsequent storage in lipid droplets results in a metabolic switch toward fatty acid oxidation (FAO) *via* STAT3 and CPT1, thus decreasing CD8^+^ T cell proliferation, survival, and overall effector function, but increasing susceptibility to ferroptosis and lipotoxicity. In contrast, T_reg_ cells use FAO and oxidative phosphorylation to sustain their immunosuppressive phenotype in a FoxP3-dependent manner through downregulation of GLUT1 and the phosphoinositide 3-kinase-Akt-mammalian target of rapamycin complex 1 (mTORC1) pathway. Lipid accumulation in DCs is mediated by MSR1/CD204 as well as in an XBP1-dependent manner, and it interferes with TLR stimulation and proliferation of DCs. Furthermore, accumulated lipids impair antigen processing and cross-presentation by HSP70 resorption. Neutrophils accumulate lipids *via* CD36/FATPs and produce leukotrienes *via* ALOX5. Lipid droplets are then transferred to metastasis-initiating tumor cells, where they facilitate survival. Natural killer (NK) cells show an impaired metabolic profile characterized by PPAR-α/δ-driven lipid accumulation and a decrease in phosphoinositide 3-kinase-Akt-mTORC1 signaling. This metabolic shift results in decreased secretion of effector cytokines, granzymes, and perforins, as well as decreased tumor cell lysis. Tumor-associated macrophages (TAMs) increase lipid uptake and storage, as well as PPAR-γ signaling *via* FAO and STAT6, thus increasing the secretion of tumor promoting and anti-inflammatory factors, and supporting angiogenesis and metastasis. Abbreviations: Akt, Akt serine/threonine kinase 1; ALOX5, arachidonate 5-lipoxygenase; ARG1, arginase 1; CCL22, chemokine (C-C motif) ligand 22; CD, cluster of differentiation; CPT1, carnitine palmitoyltransferase 1; DC, dendritic cell; FAO, fatty acid oxidation; FATP, fatty acid transport protein; FoxP3, forkhead box P3; GLUT1, glucose transporter 1; HSP70, heat shock protein 70 kDa; IFN-γ, interferon γ; IL, interleukin; MHC, major histocompatibility complex; MMP9, matrix metallopeptidase 9; MSR1, macrophage scavenger receptor 1; mTORC1, mechanistic target of rapamycin kinase complex 1; NO, nitric oxide; PD-1, programmed cell death protein 1; PD-L1, programmed cell death 1 ligand 1; PPAR, peroxisome proliferator activated receptor; STAT, signal transducer and activator of transcription; TAM, tumor-associated macrophage; TME, tumor microenvironment; T_reg_ cells, regulatory T cells; XBP1, X-box binding protein 1. Parts of the figure were drawn by using original or modified pictures from Servier Medical Art. Servier Medical Art by Servier is licensed under a Creative Commons Attribution 3.0 Unported License (https://creativecommons.org/licenses/by/3.0/).

Lipids are a diverse class of biomolecules with multiple important functions, such as serving as structural components of cell membranes, producing energy, and transducing intracellular and intercellular signals. Structural lipids, including phospholipids, sphingolipids, and cholesterol, are the major components of the plasma membrane, whereas fatty acids provide a basis for cellular bioenergetics through the β-oxidation pathway. Furthermore, several lipid molecules bind intracellular receptors, including peroxisome proliferator-activated receptors (PPARs) and sterol regulatory element-binding protein (SREBP), and subsequently regulate the transcription of genes involved in energy homeostasis and inflammation^23^. Several lines of evidence suggest that lipids are enriched in the TME and are required for immune cells to meet energy demands. However, certain lipid species in the TME cause immunosuppression, and promote cancer survival and metastasis^14,23,24^. In this section, we discuss the complicated immunosupportive or immunosuppressive roles of lipid species, such as fatty acids, cholesterol, and oxLDL (**Figure 1**).

### T cells

T cells are important mediators of anti-tumor immunity. CD8^+^ cytotoxic T lymphocytes directly or indirectly kill cancer cells through releasing effector cytokines (such as IFNγ, TNFα, granzymes, and perforin) and through death receptor-mediated contact-dependent mechanisms. After activation, T cells undergo a metabolic switch by increasing the rates of glycolysis and glutaminolysis to meet the high energy demands^25^. However, concentrations of glucose in the TME are very low, and T cells must compete with cancer cells for this scarce nutrient^26,27^. To that end, T cells increase their reliance on lipids for energy production^24^. Promotion of fatty acid β-oxidation through treatment with the PPARα agonist fenofibrate improves CD8^+^ T cell multifunctionality in a mouse melanoma model and delays tumor progression^24^. Furthermore, bezafibrate, a pan-agonist for all PPAR isoforms, increases fatty acid β-oxidation, oxidative phosphorylation, glycolysis, and CD8^+^ T cell survival, and restores CD8^+^ T cell effector function in combination with anti-PD-L1 therapy in a sarcoma mouse model, *via* upregulation of carnitine palmitoyltransferase I (CPT1) α and B cell lymphoma 2 (BCL2)^28^. Moreover, activation of PPARα and PPARβ/δ *via* the tool compound GW501516 increases CPT1α expression and fatty acid β-oxidation, which are accompanied by enhanced production of IFNγ and prolonged survival of mice in an adoptive cell therapy model^29^.

However, excessive lipid enrichment in the TME causes CD8^+^ T cell exhaustion. Tumor-infiltrating CD8^+^ T cells adapt to the lipid-rich TME by upregulating the expression of lipid transport proteins, such as CD36, which imports fatty acids including long-chain fatty acids (LCFA)^14,30^. The intracellular accumulation of LCFA drives CD8^+^ T cell dysfunction by inducing lipotoxicity. This LCFA-induced immunosuppression is further enhanced by the downregulation of very long-chain acyl-CoA dehydrogenase, an enzyme required to metabolize fatty acids, thus further increasing LCFA accumulation and exacerbating lipotoxicity^30^. Beyond fatty acids, CD36 is required to import oxLDL, a lipid species abundant in the TME^14^. OxLDL uptake into tumor-infiltrating CD8^+^ T cells promotes lipid peroxidation and ferroptosis of exhausted CD8^+^ T cells. Overexpression of the glutathione peroxidase GPX4, an antioxidant defense enzyme, enhances anti-tumor CD8^+^ T cell function^14^. One intriguing question is which molecular pathways drive tumor-infiltrating CD8^+^ T cells to adapt to the glucose-poor and lipid-rich TME. The interaction between PD-1 and its ligand PD-L1 inhibits glycolysis and promotes fatty acid β-oxidation in CD8^+^ T cells^26,31,32^, and glycolysis inhibition suppresses CD8^+^ T cell effector function^33–35^. PD-1 ligation activates signal transducer and activator of transcription 3 (STAT3), which in turn mediates the metabolic switch from glycolysis to fatty acid β-oxidation in CD8^+^ T cells in a mouse model of spontaneously developed mammary tumors^32^.

Another subset of immune cells, CD4^+^ FoxP3^+^ regulatory T (T_reg_) cells, despite being functionally distinct from CD8^+^ T cells, require fatty acid β-oxidation to exert their immunoregulatory function^36,37^. FoxP3, the key transcription factor of T_reg_ cells, promotes the expression of genes involved in oxidative metabolism at the expense of genes associated with glycolysis^38,39^. FoxP3 protects T_reg_ cells against lipid enrichment-mediated lipotoxicity by enhancing fatty acid β-oxidation and thereby decreasing fatty acid accumulation^40^.

Beyond fatty acids, cholesterol is also enriched in the TME, and tumor-infiltrating CD8^+^ T cells adapt to the cholesterol enrichment by increasing their uptake of cholesterol beyond that in splenic CD8^+^ T cells^14^. An increase in cholesterol in CD8^+^ T cells results in endoplasmic reticulum stress and T cell dysfunction^41^, a phenotype similar to that of CD8^+^ T cells deficient in synthesizing sphingolipids^42^. Plasma membrane cholesterol promotes T cell receptor clustering and enhances immunological synapse formation^43^. Genetic deficiency or pharmacological inhibition of acyl-CoA:cholesterol acyltransferase 1 (ACAT1), a key enzyme catalyzing cholesterol esterification, enhances the effector function and proliferation of CD8^+^ T cells and limits melanoma growth as well as metastasis in mice, owing to an increase in plasma membrane cholesterol.

### Natural killer (NK) cells

NK cells are part of the innate immune system and are potent producers of cytokines, including IFNγ and TNFα. Similarly to CD8^+^ cytotoxic T lymphocytes, NK cells kill malignant cells through the release of cytotoxic agents, such as perforins and granzymes, or through death receptor-ligand engagement^44,45^. The influence of dietary lipids on NK cell effector function has been studied intensively. Obesity is inversely correlated with NK cell proliferation and cancer cell cytolytic activity in both humans and mouse models^46–48^, thus implying that NK cell effector function is inhibited by lipids. Prostaglandin E is a physiologically active lipid produced by thyroid cancer cells^49^. Cancer cell-derived prostaglandin E inhibits NK cell effector cytokine production and suppresses the cytolytic activity of NK cells. Inhibition of the prostaglandin E_2_ receptor EP4 by RQ-15986 has been found to rescue the effector function of NK cells and to inhibit metastasis in a mouse model of metastatic breast cancer^50^. Furthermore, NK cells in diffuse large B cell lymphoma undergo metabolic and transcriptional reprogramming characteristic of an increase in lipid metabolism. Exposure of NK cells to a cocktail of lipids impairs NK cell function, similarly to the NK cell dysfunction observed in the lymphoma environment^51^. PPARα and PPARβ/δ increase the expression of the lipid transport protein CD36 and low-density lipoprotein receptor, thus increasing lipid uptake and accumulation in NK cells. Lipid accumulation causes a dysfunctional NK cell phenotype characterized by decreased production of effector cytokines and tumor cell lysis. The CPT1 inhibitor etomoxir restores the cytotoxicity of NK cells^52^. Similarly to PPAR, SREBP family transcription factors are conventionally known as master regulators of lipid homeostasis. SREBPs play essential roles in interleukin-2 (IL-2) and IL-12-induced metabolic reprogramming of NK cells, independently of their role in regulating lipid biosynthesis. NK cells require SREBP to elevate glycolysis and engage the citrate-malate shuttle, thereby producing IFNγ

### Tumor-associated macrophages (TAMs)

TAMs are present in many types of tumors, and their abundance is positively correlated with cancer progression and poor clinical outcomes^54–58^. Macrophages are broadly classified into pro-inflammatory (M1) and anti-inflammatory (M2) macrophages, although they display a broad spectrum of intermediate phenotypes between M1 and M2 macrophages^59,60^. This classification echoes the early definition of “classically activated macrophages” and “alternatively activated macrophages”^61–63^. Metabolites in the TME, such as lactate and fatty acids, promote the differentiation of TAMs into an M2-like phenotype^64,65^. TAMs inhibit anti-tumor immune responses through the secretion of IL-10^66,67^ and TGFβ^68,69^, and the recruitment of T_reg_ cells *via* the chemokine CCL22^70^. They also promote angiogenesis by secreting vascular endothelial growth factor^71–73^ and promote metastasis by digesting extracellular matrix proteins^74–77^. The role of fatty acid β-oxidation in IL-4-driven M2 macrophage differentiation remains debated. Some studies have suggested that TAMs increase CD36 expression to import lipids for energy production. Fatty acid β-oxidation promotes reactive oxygen species production and IL-4-STAT6 activation, and is critical for TAM polarization. In addition, CD36-dependent uptake of triacylglycerol, and subsequent lipolysis and oxidation, are required for M2 macrophage activation^78–80^. Moreover, uptake of LDL and oxLDL by the scavenger receptor MARCO fosters formation of lipid-loaded TAMs, which release CCL6 and thereby promote cancer cell migration^81^. In contrast, deficiency in CPT1a and CPT2 proteins, which are required for transporting long-chain fatty acids into the mitochondria for β-oxidation, do not affect IL-4-driven M2 macrophage differentiation^82^. Regardless of the complicated roles of fatty acid β-oxidation in IL-4-driven M2 macrophage differentiation, unsaturated fatty acids such as oleate promote the immunosuppressive and pro-tumor phenotype of TAMs through lipid droplet-dependent mechanisms^83^. In addition, SREBP1-dependent production of anti-inflammatory fatty acids contributes to the resolution of TLR4-mediated inflammation^84^, and Caveolin-1 participates in LXR-dependent cholesterol efflux and mediates anti-inflammatory properties^85^. Similarly, ovarian cancer cells facilitate membrane cholesterol efflux in TAMs, thus resulting in lipid raft breakdown and a subsequent increase in IL-4 signaling, which fosters the immunosuppressive TAM phenotype^86^.

### Dendritic cells (DCs)

DCs are professional antigen-presenting cells with essential roles in activating antigen-specific T cells. The immunostimulatory function of DCs has been shown to be inhibited by a high-fat diet^87^. Administration of polyunsaturated fatty acids attenuates DC activation and maturation^88,89^. Intracellular lipid accumulation affects antigen presentation and subsequent activation of tumor antigen-specific T cells by DCs. Briefly, the macrophage scavenger receptor (MSR1) increases lipid uptake by DCs. Lipid-laden DCs have a diminished ability to stimulate the proliferation of antigen-specific T cells, owing to defects in antigen processing^90^. *Msr1* deficiency rescues the ability of DCs to activate antigen-specific T cells^91^. Furthermore, oxidized lipids, but not non-oxidized lipids, impair DC antigen cross-presentation^92^. Lipid peroxidation byproducts activate endoplasmic reticulum stress response factor X-box binding protein 1 (XBP1), thus resulting in lipid overloading and the inhibition of DC-mediated activation of anti-tumor T cells in metastatic ovarian cancer^93^. Oxidatively truncated lipid bodies in DCs bind heat shock protein 70 (Hsp70), a protein required for trafficking of peptide-major histocompatibility complex (MHC) class I complexes to the DC cell surface. Thus, the binding of oxidatively truncated lipid bodies to Hsp70 affects peptide-MHC trafficking to the DC cell surface and disrupts DC-mediated antigen cross-presentation in cancer^94^.

### Neutrophils

Neutrophils suppress anti-tumor immune responses and support metastasis by producing leukotrienes^95,96^. Leukotrienes are bioactive lipid species that stimulate selective expansion of a subset of cancer cells with high tumorigenic potential, thus promoting tumor cell colonization of distant tissues in a mouse model of breast cancer. The production of leukotrienes is dependent on the enzyme arachidonate 5-lipoxygenase (Alox5). Inhibition of Alox5 reverses neutrophil-dependent tumor cell metastasis. The ability of neutrophils to facilitate the initiation of metastasis is associated with the accumulation of neutral lipids. In pre-metastatic states, neutrophils decrease adipose triglyceride lipase activity, thus leading to lipid accumulation. Neutrophils subsequently transfer lipids to metastatic tumor cells through a macropinocytosis-lysosome pathway, which increases tumor cell survival and proliferative ability^97^. In addition, tumor-derived oxysterols have been reported to recruit pro-tumor neutrophils in a manner dependent on C-X-C motif chemokine receptor 2, which in turn suppresses anti-tumor immune responses and supports tumor growth^98^. Collectively, lipids mediate the bi-directional communication between tumor cells and neutrophils, and cause immunosuppression and metastasis.

## Roles of amino acids in anti-tumor immunity

**Figure 2:**
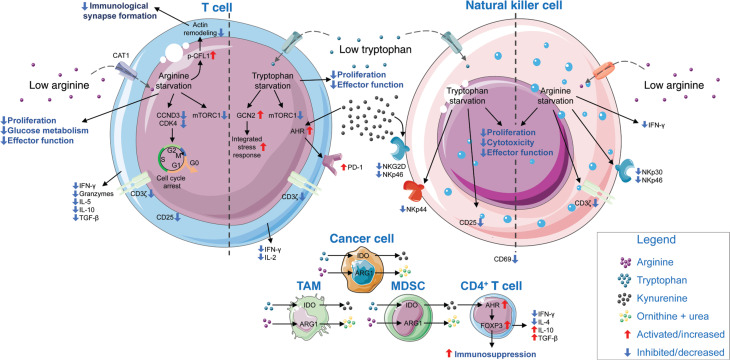
Amino acid deprivation impairs anti-tumor immunity. The TME is characterized by low arginine and tryptophan content. Cancer cells, TAMs, and MDSCs express ARG1 and IDO enzymes. ARG1 hydrolyzes arginine to ornithine and urea, and IDO catalyzes the rate-limiting reaction in tryptophan catabolism, thus ultimately resulting in the depletion of these two amino acids within the TME. Tryptophan is finally metabolized to kynurenine, which also accumulates in the TME. Arginine starvation in T cells decreases CCND3 and CDK4, thereby arresting cells in G_0_/G_1_ phase and decreasing proliferation. A decrease in immunological synapse formation is mediated by diminished dephosphorylation of CFL1 and a subsequent failure of actin remodeling, as a result of arginine starvation. Furthermore, decreases in mTORC1 signaling, glucose metabolism, and effector function are observed in T cells. Tryptophan starvation is accompanied by decreased proliferation and effector function, as well as cytokine production in T cells. Subsequently, the integrated stress response *via* GCN2 increases, and PD-1 expression is stimulated *via* kynurenine binding aryl hydrogen receptor (AHR). In NK cells, arginine starvation results in decreased proliferation, cytotoxicity, and effector function *via* downregulation of NKp30, NKp46, INF-γ, and CD3ζ. Tryptophan starvation decreases NK cell anti-tumor function by decreasing the expression of NKp44, CD25, and CD69, whereas excessive kynurenine triggers decreased expression of NKG2D and NKp46. In addition, excessive kynurenine increases the proportion of CD4^+^ CD25^+^ FOXP3^+^ regulatory T cells *via* AHR binding. Abbreviations: AHR, aryl hydrocarbon receptor; ARG1, arginase 1; CAT1, high affinity cationic amino acid transporter 1; CCND3, cyclin D3; CD, cluster of differentiation; CDK4, cyclin-dependent kinase 4; CFL1, cofilin 1; GCN2, general control nonderepressible 2; IDO, indolamine-2,3-dioxygenase; IFN-γ, interferon γ; IL, interleukin; MDSC, myeloid-derived suppressor cell; mTORC1, mechanistic target of rapamycin kinase complex; NK, natural killer; NKG2D, natural killer group 2D; NKp, natural killer protein; PD-1, programmed cell death protein 1; TAM, tumor-associated macrophage; TGF, transforming growth factor; TIM-3, T-cell immunoglobulin and mucin-domain containing-3; TME, tumor microenvironment. Parts of the figure were drawn by using original or modified pictures from Servier Medical Art. Servier Medical Art by Servier is licensed under a Creative Commons Attribution 3.0 Unported License (https://creativecommons.org/licenses/by/3.0/).

Amino acids serve not only as building blocks for protein synthesis but also as precursors for many metabolites and signaling molecules involved in numerous intracellular and intercellular signal transduction pathways. Therefore, amino acid metabolism is essential for cell proliferation, survival, and function. More than 30 years ago, Chuang et al.^99^ demonstrated that deficiency in amino acids, including arginine, glutamine, leucine, threonine, and tryptophan, inhibits T cell proliferation. This study was followed by many publications in recent decades highlighting the importance of amino acids in T cell proliferation, effector function, and differentiation^100,101^. The availability of amino acids in the TME is substantially different from that in non-tumor tissues^102^, thus prompting the question of whether amino acids and their metabolites regulate T cell exhaustion in the TME. Whereas variations in certain amino acids, e.g., glutamine, are probably based on the increased uptake by cancer cells, concentrations of these nutrients fluctuate depending on the tumor type. However, one common feature of several cancer entities is the upregulation of the arginine and tryptophan degrading enzymes arginase and IDO, respectively^103^. Therefore, in this part of the review, we focus on arginine and tryptophan as examples to discuss the roles of amino acids in shaping the immunosuppressive nature of the TME (**Figure 2**).

### Arginine

Arginine is a basic proteinogenic amino acid involved in various metabolic pathways, including the synthesis of nitric oxide, polyamines, and collagen^115–118^. Arginase 1 breaks down arginine through several intracellular and extracellular mechanisms: (i) cells increase uptake of arginine through the cationic amino acid transporter CAT2B, and arginine is then hydrolyzed by intracellular arginase 1^119^, or (ii) cells secrete arginase 1 into the extracellular compartment, where it hydrolyzes free arginine^120,121^. Arginase 1 breaks arginine down into ornithine and urea^104^. Ornithine is a precursor of polyamines such as putrescine, spermine, and spermidine, which are necessary for cell proliferation^104^.

#### T cells

Arginine plays an essential role in T cell activation and proliferation. The proliferation of both murine and human T cells is completely inhibited when T cells are cultured in arginine-free medium^110,122,123^. Comparable results have been obtained when regular cell culture medium is supplemented with arginase, whereas T cell proliferation is restored by the addition of an arginase inhibitor^110,124,125^. Furthermore, murine T cell proliferation is rescued in arginase-treated medium in the presence of the neutral amino acid citrulline, which is used as a substrate for the enzymes ASS and ASL to synthesize arginine^125^. In contrast, human T cells are not able to synthesize arginine intracellularly, owing to insufficient ASS expression in the absence of arginine^122,126^. The gene encoding the ASS protein is argininosuccinate synthetase 1 (*ASS1*). *ASS1* is a direct target of the transcription factors activating transcription factor 4 (ATF4) and CCAAT/enhancer binding protein β (C/EBPβ). Despite the presence of ATF4 and C/EBPβ, *ASS1* is not expressed in human T cells stimulated in the absence of arginine. Arginine starvation induces genome-wide chromatin compaction and increases H3 lysine-9/lysine-27 trimethylation, thus decreasing DNA accessibility, and disrupting ATF4 and C/EBPβ binding at target genes^122^. Because of their inability to synthesize arginine intracellularly, human T cells depend on the uptake of arginine from the extracellular space, a process mediated by cationic amino acid transporter 1^127^. Blocking this CAT-mediated arginine transport significantly decreases T cell proliferation and survival^126,128^.

Arginine deprivation affects T cell proliferation through multiple mechanisms, such as cell cycle arrest, decreased CD3ζ chain expression, insufficient T cell receptor signaling, decreased expression of IL-2 receptor α (CD25) and effector cytokines, and impairment of T cell metabolic fitness. Arginine increases cyclin D3 expression at the transcriptional, posttranscriptional, and translational levels^129^. Arginine starvation decreases the expression of cyclin D3, thus resulting in cell cycle arrest in G_0_-G_1_ phase^125,129^. In addition, arginine deficiency decreases the expression of the CD3ζ chain on T cells^123,124^, thereby resulting in insufficient TCR signaling to activate T cells^130^. T cells with diminished expression of CD3ζ chain have been found in tumors of patients with non-small cell lung carcinoma and a murine Lewis lung carcinoma (3LL) model, and are accompanied by elevated arginase expression in tumor cells and CAT-2B-mediated arginine uptake in arginase-expressing tumor-associated myeloid cells^120^. Interestingly, this arginine deprivation-mediated inhibitory effect on CD3ζ chain expression appears to be specific to arginine, because the depletion of other amino acids, such as glutamine, glycine, leucine, and lysine, does not change the CD3ζ chain expression level^123^. The efficient formation of the immune synapse is a prerequisite for signal transmission between antigen presenting cells and T cells^131^. The dephosphorylation of the actin-binding protein cofilin is required to induce remodeling of the actin cytoskeleton in T cells^132^. Arginine deprivation increases cofilin phosphorylation in T cells, thus impairing formation of the immunological synapse^133^. Furthermore, phorbol 12-myristate 13-acetate (PMA) and ionomycin-induced T cell activation are impaired by arginine deprivation. Because PMA and ionomycin bypass the requirement for CD3ζ chain to activate T cells, arginine depletion appears to impair T cell activation not only by affecting the CD3ζ chain-associated T cell receptor proximal signaling components but also potentially by inhibiting the distal signaling components^124^. Moreover, the expression of IL-2 receptor α chain significantly decreases under arginine deprivation, thereby indicating that arginine is indispensable for IL-2 signaling—a key signaling pathway required for T cell proliferation^123,124^. Beyond IL-2 receptor α, cytokines including IFNγ, TNFα, IL-5, and IL-10, which play important roles in T cell differentiation and effector function, are produced at low levels by T cells cultured in the absence of arginine^123,133^. Arginine deprivation impairs glycolysis, the pentose phosphate pathway, the tricarboxylic acid cycle, and mTORC1 activity^122,125^. In line with these findings, elevated arginine concentrations significantly increase the survival of human and murine CD8^+^ T cells, and the anti-tumor activity of CD8^+^ T cells in a B16 melanoma mouse model. This enhanced survival is accompanied by a shift in metabolic status from glycolysis toward mitochondrial respiration in the presence of high concentrations of arginine. Furthermore, high concentrations of arginine promote T cell differentiation into a central memory T cell-like phenotype, thus favoring the long-term persistence of anti-tumor CD8^+^ T cells^128^.

Because arginine plays essential roles in T cell activation and proliferation, inhibition of arginase has led to enthusiasm for reviving arginase-mediated dysfunctional T cells. For example, inhibition of PMN-derived arginase increases human T cell proliferation and induces higher levels of production of effector cytokines, such as IFNγ, IL-9, and IL-17. Furthermore, inhibition of arginase increases the expression of CD25 and re-expression of CD28 after the initial stimulation-induced downregulation^124,134,135^. Of note, the inhibition of arginase activity also reverses the anergic state of T cells in patients with multiple myeloma^136^.

#### NK cells

Similarly to T cells, NK cells require arginine for optimal proliferation. Arginine deprivation impairs the proliferation of murine and human NK cells induced by multiple stimuli, including IL-2 and PMA/ionomycin^110,137,138^. Compared with T cells, NK cells are less sensitive to low arginine levels. For example, T cells require arginine at a concentration of 20 μM to achieve half maximal proliferation, whereas 2 μM arginine is sufficient for NK cell half maximal proliferation^138^. Notably, NK cell viability is not affected by arginine starvation^138^. Similarly to that on T cells, ζ chain expression on NK cells is decreased by arginine starvation, thus impairing downstream signal transduction and decreasing NK cell cytotoxicity^137^. One report has shown that NK cell granule exocytosis and cytotoxicity are independent of extracellular arginine^138^. Despite the contradictory description of the influence of arginine deprivation on NK cytotoxicity, one commonality among reports is that arginine deprivation impairs IFNγ expression in NK cells through a post-transcriptional mechanism^137,138^. Additional evidence supporting the inhibitory role of arginine deprivation on NK cells is that arginase activity has been detected in damage-associated molecular patterns derived from mitochondrial preparations (MitoDAMPs)^139^. MitoDAMPs impair IFNγ secretion by NK cells and decrease the expression of the NK cell activating receptor NKG2D. The inhibitory effects of MitoDAMPs are reversed by the addition of extracellular arginine or an arginase inhibitor^139^. MitoDAMPs are detectable in cancers^140,141^, thus implying that MitoDAMPs may impair NK cell function by depleting arginine in the TME.

### Tryptophan

Tryptophan is an essential amino acid. Because mammalian cells are incapable of synthesizing tryptophan, dietary intake is the major source. Tryptophan is crucial for protein synthesis, and maintaining cell growth and proliferation, and is involved in the biosynthesis of the neurotransmitter serotonin and the hormone melatonin. More than 95% of free tryptophan serves as a substrate for the kynurenine pathway, in which tryptophan is degraded to nicotinic acid, the precursor of nicotinamide adenine dinucleotide, a key coenzyme in energy metabolism and redox reactions^143–145^ and many tumor cells, including melanomas, and cervix, kidney, non-small lung and colorectal carcinomas^146–148^. High expression of the tryptophan degrading enzyme IDO1 decreases the abundance of tryptophan in patients with breast cancer, colorectal cancer, head and neck cancer, prostate cancer, and lung cancer^143^. Reciprocally, kynurenine levels are elevated in the plasma of patients with cancer^143,149^.

#### T cells

T cells show less proliferation when cultured in tryptophan-free medium, IDO-conditioned medium, or medium supplemented with kynurenine and picolinic acid than in regular tryptophan-replete medium^117,144,147,150^. Of note, the effects of tryptophan deprivation on T cell proliferation vary across experimental conditions. For example, the anti-CD3 or concanavalin A-driven proliferation of murine CD8^+^ T cells is inhibited when cells are cultured with kynurenine and low concentrations of tryptophan, and this effect is accompanied by diminished secretion of the cytokines IL-2 and IFNγ^150^. In contrast, murine CD8^+^ T cells proliferate and produce cytokines normally when stimulated with PMA and ionomycin^144,147,151^, thus suggesting that tryptophan deprivation affects T cell proliferation and cytokine production by impairing T cell receptor proximal signaling components. Furthermore, the observation that tryptophan deprivation induces an integrated stress response also depends on the experimental setting. Briefly, in the absence of amino acids, the general control nonderepressible 2 (GCN2) kinase is activated by the accumulation of uncharged tRNAs and triggers the integrated stress response^152^. Exposure of murine CD8^+^ T cells to IDO-expressing DCs activates GCN2 kinase and results in complete inhibition of CD8^+^ T cell proliferation^153^. Similarly, GCN2 kinase is activated in CD4^+^ T cells by tryptophan deprivation^117^. In contrast to the two studies described above, GCN2 kinase activation is not observed in tumor-infiltrating T cells^154^.

Beyond regulating T cell proliferation and stress responses, IDO regulates T cell differentiation. Co-culturing human CD4^+^ T cells with IDO-expressing DCs or cancer cells increases the differentiation of CD4^+^ CD25^+^ FoxP3^+^ T_reg_ cells with potent suppressor function^147,155,156^. The enhanced T_reg_ cell differentiation is accompanied by increased production of IL-10 and TGFβ, and a reciprocal decrease in IL-4 and IFNγ^150^. Inhibition of IDO suppresses T_reg_ cell differentiation, which is restored by the addition of kynurenine^155,156^. IDO-driven T_reg_ cell differentiation is dependent on the activation of the ligand-activated transcription factor AHR *via* kynurenine binding^157,158^. Kynurenine-mediated AHR activation upregulates PD-1 expression in tumor-infiltrating murine CD8^+^ T cells, thus conferring a CD8^+^ T cell exhaustion phenotype^159,160^. Furthermore, tryptophan-derived metabolites, such as kynurenic acid and xanthurenic acid, also activate AHR^161,162^ and may contribute to T_reg_ cell differentiation and CD8^+^ T cell exhaustion.

The mechanisms through which IDO inhibits T cell proliferation *in vivo* remain open to debate. One possibility is that IDO suppresses T cell responses simply by decreasing the availability of an important essential amino acid. The other possibility is that kynurenine, the product of an IDO-mediated enzymatic reaction, causes T cell inhibition. Mass spectroscopy analysis has demonstrated that intra-tumoral tryptophan concentrations are above the threshold triggering the integrated stress response^154^. Thus, the *in vivo* immunosuppressive function of tryptophan metabolism is likely not to be caused by tryptophan depletion^154^.

#### NK cells

Tryptophan metabolism also affects NK cell proliferation and effector function. Culturing NK cells in the presence of purified IDO enzyme or co-culturing NK cells with IDO-expressing DCs inhibits NK cell proliferation. This inhibitory effect is partly restored by an IDO inhibitor^143,147,163^. Similarly to that of T cells, NK cell cytotoxicity is inhibited by kynurenine. Kynurenine also decreases the expression of the NK cell activating receptor NKG2D and natural cytotoxicity triggering receptor 1 (NCR1, also known as NKp46)^164^. In contrast, NKp30-mediated cytotoxicity is unaffected by kynurenine, thus implying that the inhibitory function of kynurenine on NK cells is not dependent on the NKp30 pathway^164^. Finally, inhibition of IDO increases NK cell cytotoxicity and NK cell numbers in ovarian tumors^163,165^.
